# Joint effects of polymorphisms in the *HTRA1, LOC387715/ARMS2*, and *CFH* genes on AMD in a Caucasian population

**Published:** 2008-08-04

**Authors:** Peter J Francis, Hong Zhang, Andrew DeWan, Josephine Hoh, Michael L Klein

**Affiliations:** 1 Macular Degeneration Center, Casey Eye Institute, Oregon Health & Science University, Portland, OR; 2 Department of Epidemiology and Public Health, Yale University School of Medicine, New Haven CT

## Abstract

**Purpose:**

To estimate the joint effects of single nucleotide polymorphysms (SNPs) in the genes *complement factor H* (*CFH)*, *HtrA serine peptidase 1* (*HTRA1),* and *age-related maculopathy susceptibility 2* (*LOC387715/ARMS2)* in a Caucasian age related macular degeneration (AMD) case-control cohort.

**Methods:**

We genotyped three SNPs, rs1061170 (exon 9, *CFH*), rs11200638 (*HTRA1* promoter, −512 bp), and rs10490924 (6.6 kb upstream of *HTRA1* in *LOC387715/ARMS2*) in 333 cases with advanced AMD (choroidal neovascularization [CNV] and geographic atrophy) and 171 age-matched examined controls. Association tests were performed for individual SNPs and jointly with the *CFH* SNP Y402H. Analyses for interaction were also performed.

**Results:**

The linkage disequilibrium measure for two SNPs on 10q26, rs10490924 and rs11200638, is D'=0.8 and all four possible haplotypes of the two SNPs were detected in the samples. The allelic association test for rs11200638 on the promoter of *HTRA1* yielded p-values less than 10^−10^ for geographic atrophy, less than 10^−16^ for neovascularization, and less than 10^−19^ for the pooled phenotypes (with an odds ration [OR] of 3.973; 95% confidence interval [CI] 2.928, 5.390). Disease risk is conferred in a dosage-dependent fashion. Similar figures were observed for the *LOC387715/ARMS2* SNP. No interaction was detected between either between the 10q26 SNPs or the *CFH* SNP.

**Conclusions:**

This is the first analysis to show that the two 10q26 SNPs are not in complete linkage disequilibrium. Our studies however show that both the *HTRA1* and *LOC387715/ARMS2* SNP appear to contribute equally to disease risk (both geographic atrophy and choroidal neovascularization) with no evidence of interaction with *CFH.*

## Introduction

Several genes have now been associated with the development of age-related macular degeneration (AMD). The most consistently identified genetic variants lie within the regulators of complement activation (RCA) locus on chromosome 1, principally the gene *complement factor H* (*CFH)* [1-3], and the 10q26 chromosomal region [4-6]. Association analyses of this latter locus initially implicated the single nucleotide polymorphism (SNP) rs10490924, located in the coding sequence of a gene now known as *age-related maculopathy susceptibility 2* (ARMS2) [4-6], to be the variant most likely associated with AMD. Subsequent rigorous sequencing of the region revealed a polymorphism, rs11200638, in the promoter of the adjacent gene, *HtrA serine peptidase 1* (*HTRA1)*, some 7 kb downstream of *LOC387715/ARMS2*, to be also associated with advanced AMD [7,8]. To date, this finding has been corroborated in two other Caucasian [9,10] one central European [11], and one Japanese [12] populations.

Intriguingly, both rs10490924 and rs11200638 are in almost complete linkage disequilibrium in all cohorts examined thus far (D' >0.98) [7,12,13], limiting the ability of genetic association analyses to resolve the functional variant. Initial molecular biologic analyses suggest that the promoter SNP in *HTRA1*, a gene that encodes a heat shock serine protease found in retinal tissues, produces a change in the expression level of the gene [7,8]. Interestingly, HTRA1 expression has been reported to increase with age [14].

Most recently, *LOC387715/ARMS2* has been suggested to encode a mitochondrial-associated protein that is also found in the retina. The polymorphism which results in the substitution of serine-for-alanine at position 69 may result in misfolding of the protein [15].

In this article, we present further analyses of the 10q26 polymorphisms in a Caucasian population from the USA with advanced AMD (both geographic atrophy and choroidal neovascularization) and assess joint effects of the *HTRA1* rs11200638 SNP, the *LOC387715/ARMS2* SNP rs10490924 (A69S), and the *CFH* SNP rs1061170 (Y402H).

## Methods

### Phenotyping

Sporadic advanced cases (average age 79 years of age, range 60 to 100 years of age) and controls (average age 74, range 63 to 92 years of age, Table 1) of Northern European Caucasian descent were ascertained from the clinical practices of P.F. and M.K. Diagnosis of AMD in was based upon the presence of geographic atrophy or choroidal neovascularization (CNV; equivalent to Age-Related Eye Disease Study [AREDS] category 4) [16]. Control subjects were at least 60 years of age, with no signs of AMD (defined as no drusen larger than 63 μm in diameter; equivalent to AREDS category 1).

**Table 1:** Summary of age distributions for the four phenotypic groups

**Phenotype**	**Sample size**	**Min**	**1st quantile**	**Mean**	**Max**
Control	171	58	68	73	92
Dry	108	48	75	79	95
Dry+Wet	20	69	74	79	91
Wet	205	40	74	78	100

Counts, Hardy-Weinberg equilibrium tests, allele frequencies, genotypic association tests for three risk SNPs in each and combined cohorts.

One hundred and eight patients with geographic atrophy (GA) together with 205 patients with CNV, 20 with both GA and CNV, and 171 age-matched examined controls were ascertained. Informed consent was obtained from all participants, and the procedures used conformed to the tenets of the Declaration of Helsinki.

### Genotyping

Genotyping of rs1061170, rs10490924, and rs11200638 was performed as described previously [17]. Specifically, PCR was performed using primers designed to amplify the genomic region containing each SNP (rs10490924 forward: 5′-GGT GGT TCC TGT GTC CTT CA-3′, reverse: 5′-GGG GTA AGG CCT GAT CAT CT-3′; rs11200638 forward: 5′-CGG ATG CAC CAA AGA TTC TCC-3′, reverse: 5′-TTC GCG TCC TTC AAA CTA ATG G-3′). Following amplification, genotype determination was performed on the PCR products using either temperature gradient capillary electrophoresis (TGCE; REVEAL; SpectruMedix, State College, PA) or through direct sequencing using CEQ2000XL DNA analysis system (Beckman Coulter, Fullerton, CA).

### Statistical analyses

Hardy–Weinberg Equilibrium (HWE) χ^2^ values in the entire sample and controls alone were calculated to identify possible genotyping errors. No extreme deviations (i.e., χ^2^ > 50) were observed (Table 2). Linkage disequilibrium (LD) was measured by the D' value. For each SNP, Pearson’s χ^2^ tests with one degree of freedom for association were performed. Odds ratios (OR) and their respective confidence intervals were calculated, [18]. Both SNPHAP and PHASE were used to estimate the haplotype frequencies and to reconstruct the diplotype (haplotype pair) for each sample. Consistent results obtained by using both algorithms were taken for further regression analyses.

**Table 2:** Genotyping information and Fisher’s exact tests for association between SNPs and disease status

**SNP**	rs11200638 **(*HTRA1 promoter SNP*)**	rs10490924 **(*LOC387715/ARMS2*)**	rs1061170 **(*CFH*)**
HWE χ^2^	5.37	0.702	0.116
Genotypic /Allelic test			
GA	2.05e-8/ 6.94e-11	2.36e-9 / 9.83e-12	1.14e-6 / 4.80e-7
GA+CNV	7.86e-7 / 1.33e-8	3.97e-7 / 1.92e-8	3.98e-3 / 1.31e-3
CNV	9.82e-15 / 1.67e-17	6.39e-16 / 2.42e-17	9.60e-6 / 1.96e-6
Pooled cases	2.70e-17 / 7.55e-21	3.12e-17 / 1.10e-19	4.78e-8 / 1.52e-8
Odd Ratio (95% CI)	3.973 (2.928, 5.390)	4.671 (3.245, 6.722)	2.399 (1.768, 3.256)

Genotyping data confirming Hardy-Weinberg equilibrium for all SNPs analyzed. Fisher’s exact tests shows strong association between SNPs and advanced AMD disease status. Abbreviations: HWE=Hardy–Weinberg Equilibrium. Fisher’s exact genotypic / allelic association test for various disease status: p-value. GA=geographic atrophy, CNV=choroidal neovascularization, GA+CNV refers to those individuals with GA and CNV in one or both eyes.

Joint ORs for two SNPs (rs11200638 and complement factor H (*CFH*) Y402H, previously genotyped) were calculated using standard methods [19]. Marginal ORs and their confidence intervals for the two SNPs were calculated using logistic regression with SNPs *CFH* Y402H and rs11200638 as independent variables [19]. The standard logistic regression models for marginal and joint effect (effect of marker X_i_ controlled for marker X_j_) are formulated as logit(p)=β_0_ + β_1_X_1_ and logit(p)=β_0_ + β_1_X_1_ + β_2_X_2_, respectively. For highly correlated SNPs (rs10490924 and rs11200638), an interaction term was incorporated in the model: logit(p)=β_0_ + β_1_X_1_ + β_2_X_2_ + β_3_X_1_×X_2_.

To assess the statistical significance of the effect, the likelihood ratio test is performed. To control for confounding, the Mantel-Hanzel (M-H) test for association with two variables was used [19]. Four genotypic models were considered (Full, Recessive, Multiplicative, and Dominant) and the conventional Aikake information criterion (AIC) [20] was used to assess the fit of each model.

## Results

In our US Caucasian case-control cohort, the *HTRA1* rs11200638 SNP showed strong disease association with both advanced forms of AMD, p<10^−10^ (geographic atrophy), p<10^−16^ (neovascularization), and p<10^−20^ for pooled phenotypes. The *LOC387715/ARMS2* rs10490924 SNP showed similar levels of association. Strong association is also confirmed between disease status and the *CFH* SNP rs1061170 (Table 2).

The two 10q26 SNPs, rs11200638 and rs10490924, were also in strong linkage disequilibrium (D'=0.80), though not in complete LD as all possible diplotypes were found in the population. Table 3 shows how the four potential haplotypes produced by the two SNPs were distributed; AMD cases were approximately three times more likely to have the high risk haplotype rs10490924 ‘T’ + rs11200638 ‘A’ than controls.

**Table 3:** Haplotype frequencies with estimated standard deviations in parentheses

**Haplotype (rs10490924 + rs11200638)**	**Pooled sample**	**Case**	**Control**
GG	0.412 (0.006)	0.277 (0.006)	0.679 (0.014)
GA	0.190 (0.005)	0.229 (0.006)	0.114 (0.006)
TG	0.124 (0.007)	0.143 (0.006)	0.087 (0.014)
TA	0.273 (0.005)	0.351 (0.007)	0.119 (0.005)

Haplotype frequencies for the *LOC387715* and *HTRA1* SNPs (with estimated standard deviations in parentheses) showing the presence of all four potential haplotypes in the cohort. P-value for independence between haplotypes and disease: 1.1e-22.

Table 4 shows joint and marginal odds ratios for the *HTRA1* and *CFH* SNPs. Odds ratios of having the disease rise to almost 200 in individuals who are homozygous for the risk alleles in both genes. Similar odds ratios are observed for the *LOC387715/ARMS2* and *CFH* SNP (Table 5). When joint odds ratios were computed for the two highly correlated SNPs on 10q26 and the interaction terms considered in the regression model, the resulting confidence interval encompasses 0 and ∞ due to insufficient sample size in each cell (Table 6).

**Table 4:** Joint and marginal odds ratios (pooled cases) for rs11200638 and rs1061170

rs11200638	rs1061170			rs11200638 ** risk (adjusted for rs1061170)**
	TT	TC	CC	
GG	1	3.53	3.92	1
GA	4.45	11.69	25.98	4.30 (95% CI: 2.50, 7.39)
AA	6.33	15.55	192.71	8.13 (95% CI: 3.70, 17.87)
rs1061170 risk (adjusted for rs11200638)	1	3.17 (95% CI: 1.77, 5.66)	5.74 (95% CI: 2.75, 11.98)	

Marginal odds ratios are calculated based on a logistic regression model by assuming an additive effect of two SNPs after a logit transformation on odds ratio

**Table 5:** Joint and marginal odds ratios (pooled cases) for rs10490924 and rs1061170

rs10490924	rs1061170			rs10490924 ** risk (adjusted for rs1061170)**
TT	TC	CC
GG	1	3.59	4.38	1
GT	5.72	13.66	36.52	5.24 (95% CI: 3.08, 8.91)
TT	7.63	38.77	227.57	15.30 (95% CI: 5.75,40.73)
rs1061170 risk (adjusted for rs10490924)	1	3.32 (95% CI: 1.87, 5.91)	6.12 (95% CI: 2.92, 12.85)	

Marginal odds ratios are calculated based on a logistic regression model by assuming an additive effect of two SNPs after a logit transformation on odds ratio.

**Table 6:** Joint genotype counts in cases/controls for rs10490924 and rs1061170

	rs11200638		
rs10490924	77/74	39453	0/0
	0/5	27/117	39549
	0/2	0/5	24959

Joint genotype counts of controls/cases, sample size too small to fit regression model with interaction terms.

Haplotype analyses of these two closely located SNPs were therefore employed using SNPHAP and PHASE programs (Table 7). Under the regression framework, no interaction was detected between the 10q26 haplotypes and the *CFH* SNP (Table 8). After dropping the interaction term the effect of one controlled for the other can be estimated and tested for significance based on the likelihood ratio test (Table 9). Estimated haplotype frequencies, diplotype counts, odds ratio, and p-values for testing independ for all observed diplotypes are provided in Table 10.

**Table 7:** Tests for model fit of the effects in rs11200638 and rs1061170

**Model for rs11200638** **x rs1061170**	**PAR % (95% CI)**		**M-H test: p-value**		**LRT:** **p-value**	**AIC** **value**
rs11200638	rs1061170	rs11200638	rs1061170
Full	58.0 (46.4, 67.0)	62.8 (45.5, 74.4)	4.24e-11	2.07e-6	1.20e-2	398.3
Rec x Rec	21.6 (13.9, 28.7)	20.1 (10.2, 28.9)	1.08e-4	2.20e-3	2.12e-2	439.5
Rec x Mul	21.7 (14.4, 28.7)	75.7 (63.3, 83.8)	1.05e-4	2.11e-6	1.62e-1	425.6
Rec x Dom	22.3 (14.4, 29.4)	60.4 (43.1, 73.6)	3.73e-5	3.38e-6	4.28e-1	428.6
Mul x Rec	67.2 (59.1, 74.0)	19.8 (8.8, 28.5)	6.11e-11	3.06e-3	2.15e-2	411.7
Mul x Mul	67.4 (60.1, 73.9)	76.1 (64.9,84.1)	4.24e-11	2.07e-6	1.45e-1	397.5
Mul x Dom	67.7 (59.8, 74.2)	62.0 (45.0, 74.9)	1.03e-11	2.83e-6	7.45e-1	399.3
Dom x Rec	56.9 (45.2, 66.2)	20.7 (10.3, 29.2)	3.63e-11	1.71e-3	4.77e-2	412.5
Dom x Mul	57.5 (47.1, 66.0)	76.7 (66.1, 84.3)	2.51e-11	1.06e-6	2.47e-1	397.8
Dom x Doc	58.2 (47.4, 67.3)	62.9 (45.7, 74.9)	6.88e-12	1.80e-6	9.51e-1	400.6

Models for rs11200638 × rs1061170. Note: According to AIC value, Mul x Mul model fits the data best. Similar results for rs10490924 × rs1061170.

**Table 8:** Joint analyses of the CFH SNP and 10q26 haplotypes

rs10490924 ** + rs11200638**	rs1061170			rs10490924 ** + rs11200638 risk (adjusted for rs1061170)**
	TT	TC	CC	
GG/GG	1	2.09	4.48	1
Non GG/GG	5.44	14.4	26.86	6.49 (95% CI: 4.01, 10.50)
rs1061170 risk (adjusted for rs10490924 + rs11200638)	1	2.34 (95% CI: 1.34, 4.08)	4.90 (95% CI: 2.40, 10.00)	

The haplotype information for 10q26 gene is estimated using software PHASE. By treating the haplotype GG (formed by high risk alleles G and G at rs10490924 and rs11200638) as a new “allele”, we studied the joint and main effects of CFH SNP-10q26 haplotypes as in Table 3. Here a recessive mode of inheritance for 10q26 gene is assumed so that the gentotypes are classified to GG/GG and non GG/GG.

**Table 9:** Estimated frequencies and their standard deviations of haplotypes for rs10490924 and rs11200638

**Haplotype (rs10490924 +rs11200638)**	**Pooled sample**	**Case**	**Control**
GG	0.412 (0.006)	0.277 (0.006)	0.679 (0.014)
GA	0.190 (0.005)	0.229 (0.006)	0.114 (0.006)
TG	0.124 (0.007)	0.143 (0.006)	0.087 (0.014)
TA	0.273 (0.005)	0.351 (0.007)	0.119 (0.005)

Presented in parentheses are estimated standard deviations. All the results were obtained by software SNPHAP using joint genotypes of SNPs rs10490924 and rs11200638.

**Table 10:** Counts, odds ratio, and p-value for testing independence for all observed diplotypes (rs10490924 x rs11200638)

	**GG/GG**	**GG/GA**	**GG/TG**	**TG/TG**	**GG/TA**	**GA/TA**	**TG/TA**	**TA/TA**
Case	134	1	0	0	11	33	5	0
Control	89	6	5	2	37	139	73	13
Odds ratio	1	---	---	---	5.06 (95% CI: 2.45,10.45)	6.34 (95% CI: 3.99,10.09)	21.98(95% CI: 8.54,56.55)	---
P-value	---	0.02	0.01	0.16	2.62e-6	1.51e-16	2.41e-18	1.16e-5

Odds ratios and their CIs are based on logistic regression models. Where counts are less than 6, ORs are ignored; p-values are for Fisher’s exact tests. The phase-known diplotypes and estimated diplotypes were pooled, which were sufficient for haplotype analyses. P-value for testing independence of disease and all diplotypes is 4.76e-24.

## Discussion

Our data confirm the association between advanced AMD (both geographic atrophy and CNV) and the 10q26 SNPs rs11200638 (*HTRA1* promoter) and rs10490924 (*LOC387715/ARMS2* A69S), independent of the *CFH* Y402H polymorphism. In all previous papers which examined linkage disequilibrium in the 10q26 region, these two SNPs have been reported to be in almost complete linkage disequilibrium [7,13]. This was not the case in our population where the D' between the SNPs was 0.80. This is a critical finding as it does indicate that other studies using larger populations may be able to determine which of the two 10q26 SNPs may be contributing most to disease status. The number of individuals in our study was too small to permit this. Odds ratios of having advanced AMD, when adjusted for the *CFH* SNP, were not significantly different for either of the 10q26 SNPs genotyped in our cohort. Each of the two SNPs also confers similar odd ratios when combined with the *CFH* SNP. Neither SNP was more clearly associated with disease status nor with advanced AMD phenotype though arguably if our cohort is enlarged substantially it might be possible to distinguish which SNP contribute the most risk.

Previous studies [21] have not identified gene-locus interaction between *CFH* and the region on chromosome 10q26. Our analyses are in agreement with this. Thus it seems likely that *CFH* and 10q26 contribute independently to AMD development, at least at the genomic level.

There remains an enormous amount to be learned about the genetic etiology of AMD. Nonetheless, there is little question that either *HTRA1* and/or *LOC387715/ARMS2* or a 10q26 haplotype that expands more than two SNPs play a crucial role in determining the advanced AMD phenotypes. Functional consequences from the polymorphisms of *LOC387715/ARMS2* or *HTRA1,* or even *CFH,* have yet to be determined. Our current data indicate that in a Caucasian population from the USA, the *HTRA1* promoter SNP is strongly associated with advanced AMD but appears to exert its effect independently of *CFH*.
